# The Formation and Displacement of Ordered DNA Triplexes in Self-Assembled Three-Dimensional DNA Crystals

**DOI:** 10.1021/jacs.2c12667

**Published:** 2023-02-02

**Authors:** Yue Zhao, Arun Richard Chandrasekaran, David A. Rusling, Karol Woloszyn, Yudong Hao, Carina Hernandez, Simon Vecchioni, Yoel P. Ohayon, Chengde Mao, Nadrian C. Seeman, Ruojie Sha

**Affiliations:** † Department of Chemistry, New York University, New York, New York 10003, United States; ‡ The RNA Institute, University of Albany, State University of New York, Albany, New York 12222, United States; § School of Pharmacy and Biomedical Sciences, University of Portsmouth, Portsmouth PO1 2DT, U.K.; ∥ Department of Chemistry, Purdue University, West Lafayette, Indiana 47907, United States

## Abstract

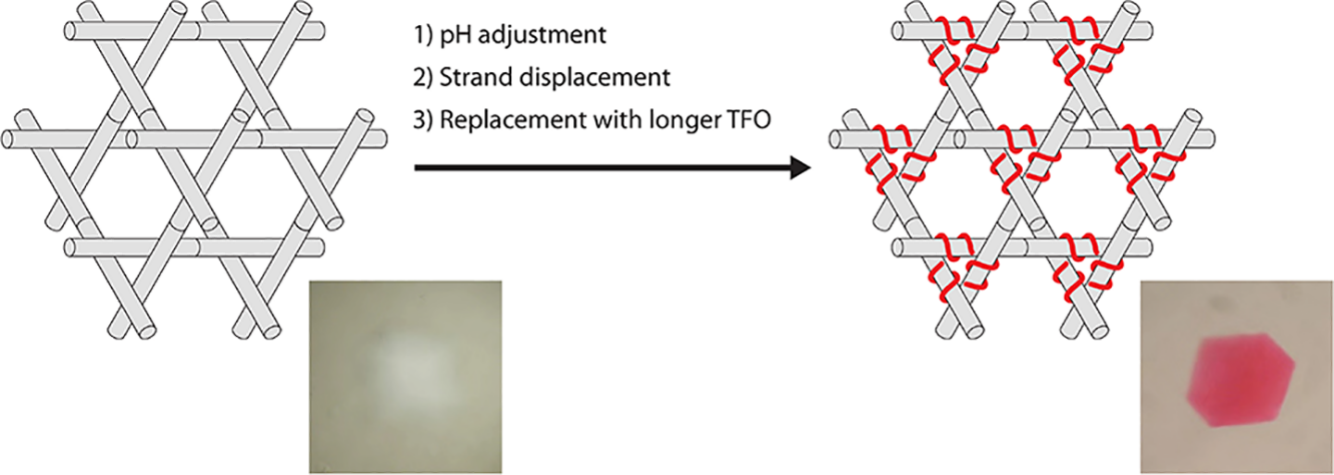

Reconfigurable structures engineered through DNA hybridization and self-assembly offer both structural and dynamic applications in nanotechnology. Here, we have demonstrated that strand displacement of triplex-forming oligonucleotides (TFOs) can be translated to a robust macroscopic DNA crystal by coloring the crystals with covalently attached fluorescent dyes. We show that three different types of triplex strand displacement are feasible within the DNA crystals and the bound TFOs can be removed and/or replaced by (a) changing the pH from 5 to 7, (b) the addition of the Watson–Crick complement to a TFO containing a short toehold, and (c) the addition of a longer TFO that uses the duplex edge as a toehold. We have also proved by X-ray diffraction that the structure of the crystals remains as designed in the presence of the TFOs.

## Introduction

**Figure 1:**
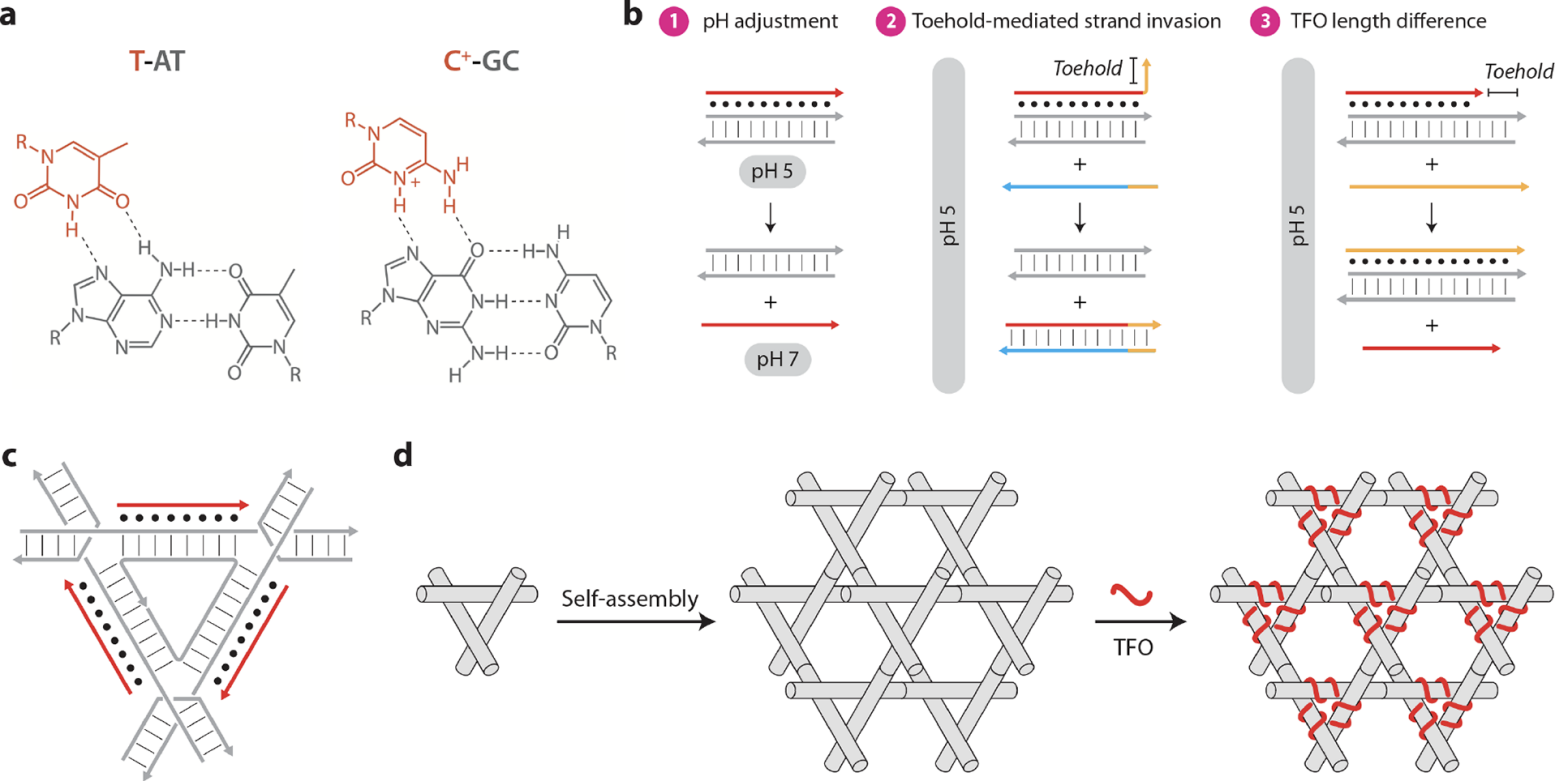
Concept and design. (a) Chemical structure of parallel T·A-T and C^+^·G-C triplets generated upon binding of a TFO to duplex DNA. (b) Schematic drawings of the three strategies for displacing/exchanging duplex-bound TFOs used in this study. (c) Schematic drawing of TFO binding to the tensegrity triangle motif. (d) Schematic drawing of the self-assembly of the DNA crystal with TFOs. TFOs are shown in red and duplex strands in gray.

**Figure 2:**
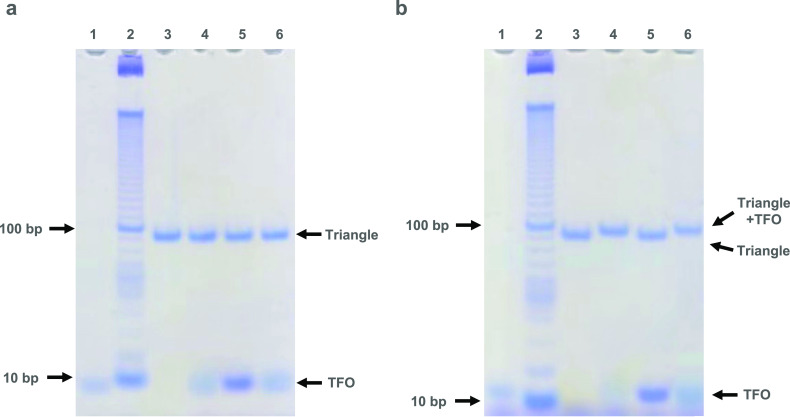
TFO strand displacement on a tensegrity triangle motif analyzed by native PAGE at different pH values. Lane 1 contains an 11-nt TFO strand, lane 2 contains a 10 bp ladder, lane 3 contains a triangle motif, lane 4 contains a triangle motif and 11-nt TFO, lane 5 contains the triangle motif with 11-nt TFO and its Watson–Crick complement, and lane 6 contains a triangle motif, 11-nt TFO, and 13-nt TFO that has the potential to displace the bound 11-nt TFO. (a) Samples were prepared and run in pH 7 TA-Mg buffer and revealed the binding and displacement of the TFOs. (b) Samples were prepared and run in pH 5 TA-Mg buffer and revealed no binding of TFOs to the triangle.

**Figure 3:**
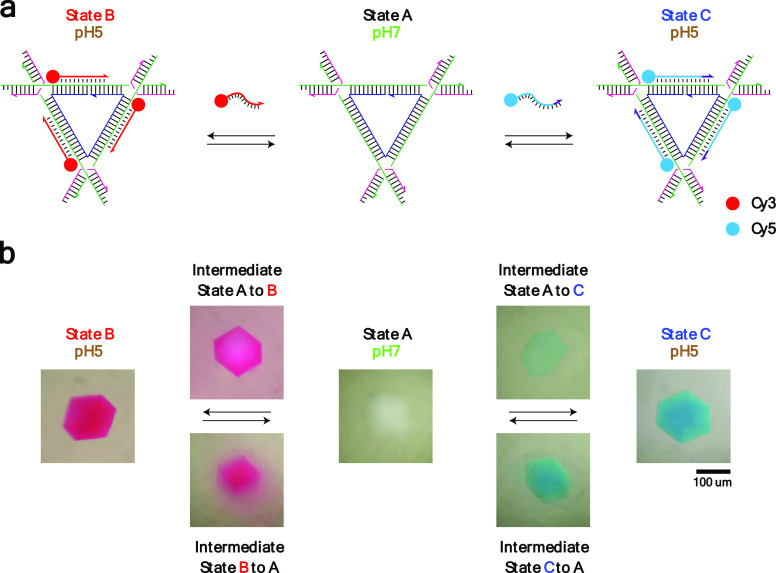
pH-induced TFO displacement. (a) Schematic drawing of the strategy: A DNA tensegrity triangle (state A) is bound with a Cy3-bearing 11-nt TFO or Cy5-bearing 13-nt TFO (state B or C, respectively) at pH 5. The binding is reversed by increasing the pH to 7 (state A). (b) Crystal images: Corresponding to the scheme shown above, pH-induced TFO displacement from state B (red) to state A (clear) and from state C (blue) to state A (clear) was achieved by raising the pH from 5 to 7. The processes are reversed by decreasing the pH from 7 to 5. Crystal images above and below the transition arrows are intermediate states. The difference of the color pattern of those crystals indicates that the displacement and binding of TFOs start at the edge and diffuse into the center of the crystals.

DNA nanotechnology enables us to self-assemble various nanoscale structures by programming DNA sequences.^1−6^ Both structural and dynamic applications are achieved with reconfigurable DNA nanostructures constructed using DNA programmability.^7,8^ Dynamic DNA nanostructures have been shown to respond to environmental stimuli such as pH, chemical stimuli such as ionic conditions, physical stimuli such as light, and biological stimuli such as other macromolecules. The use of triplex-forming oligonucleotides (TFOs) in DNA nanotechnology provides additional levels of control by exploiting combined stimuli, such as pH-induced TFO binding, light-triggered crosslinking of DNA motifs, and strand displacement-induced reconfiguration.^9^ Triplexes are generated by the binding of a TFO within the duplex major groove by specific Hoogsteen base pairing interactions with exposed groups on the duplex base pairs (Figure 1a).^10^ A homopyrimidine strand binds in a parallel orientation to a homopurine strand of the duplex, with thymine and protonated cytosine recognizing AT and GC base pairs, respectively.^11^ Here, we explore dynamic control of TFO binding and removal using three separate strategies (Figure 1b): First, triplexes containing C^+^·GC triplets can only form at low pH (typically below 6) due to the requirement for cytosine protonation^12,13^ and, thus, by increasing the pH to 7, the TFO can be removed from the duplex (Figure 1b-1). Second, the incorporation of a short single-stranded toehold to the termini of the TFO allows its removal by toehold-mediated strand invasion upon addition of its Watson–Crick complement. Importantly, this leaves the underlying duplex intact and would allow, for example, the binding of a second TFO to the same target sequence (Figure 1b-2). Third, a shorter TFO sequence can be directly replaced by a longer TFO sequence that has more Hoogsteen complementarity to the duplex edge, which acts as the toehold in a triplex context (Figure 1b-3).

We also could apply these strategies to the formation and displacement of ordered TFOs within three-dimensional (3D) DNA crystals self-assembled from the tensegrity triangle motif (Figure 1c).^14−16^ The tensegrity triangle is a robust motif composed of three double helices directed along linearly independent vectors.^14^ By tailing the helices with complementary single-stranded overhangs, each triangle can associate with six others, yielding rhombohedral DNA crystals.^15^ We have previously demonstrated that TFOs can bind to a homopurine–homopyrimidine target sequence embedded within or between the tiles of a tensegrity triangle crystal.^17−19^ There is a rough minimum length for the TFOs that bind to the duplex region within the motifs, somewhere around nine nucleotide pairs.^20,21^ Our standard 2-turn/edge tensegrity triangle component has 7 nucleotides between crossover junctions. Unless we wish to have the triplex bind through junctions, the edges of this triangle are too short. Consequently, we have used a larger triangle, with three turns per edge, containing 17 nucleotide pairs between crossover points within each triangle. Except for its sequence, this 3-turn/edge triangle is a known molecular structure, and we have established its crystal structure previously.^16^ We modified the sequences of the edges to include a TFO-binding site (Table S1). Further, the triangle motif is 3-fold symmetric, with all three edges containing the same sequences (thus, one triangle can bind three TFOs). The homopurine–homopyrimidine segment in the edges of the tensegrity triangle is complementary to the TFO strand in the sense of triplex complementarity; the TFO is a homopyrimidine strand that binds to the major groove of this duplex. The idea behind using DNA triplex formation as the basis of an ordered guest system is that there are multiple attachment points for the triplex strand, in principle, one Hoogsteen pair per nucleotide pair. TFOs can be bound to the edges of the motifs, which can then self-assemble into 3D DNA crystals, or as in this study, the TFOs can be soaked into the crystal solution to yield ordered triplex formation within the crystalline lattice with little disruption of the underlying duplex DNA (Figure 1d).

## Results

**Figure 4:**
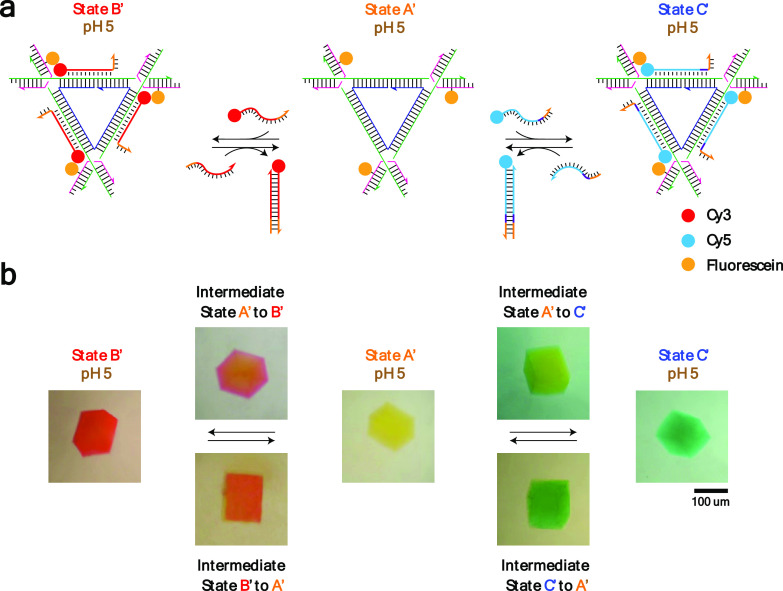
Toehold-mediated strand invasion-induced TFO displacement. (a) Schematic drawing of the strategy: A fluorescein-labeled DNA tensegrity triangle (state A′) is bound with Cy3-bearing 11-nt or Cy5-bearing 13-nt TFOs containing toeholds (state B′ or C′, respectively) at pH 5.0. The binding is reversed by adding the Watson–Crick complementary strands of toehold-bearing 11-nt or 13-nt TFOs at pH 5.0. (b) Crystal images: Corresponding to the scheme shown above, toehold-mediated strand invasion induced TFO displacement from state B′ (coral) to state A’ (yellow) and from state C′ (green) to state A’ (yellow) by adding Watson–Crick-complementary strands of 11-nt or 13-nt TFO strands, respectively. It is reversible by adding more Cy3-bearing 11-nt or Cy5-bearing 13-nt TFOs. The triangles in state A′ have fluorescein attached to them, so there is a yellow cast to the crystals; the coral color in state B′ is the mixture of yellow and red, and the green color in state C′ is the mixture of yellow and blue. Crystal images of intermediate states indicate that the displacement and binding of TFOs follows a similar pathway to that seen in Figure 3.

**Figure 5:**
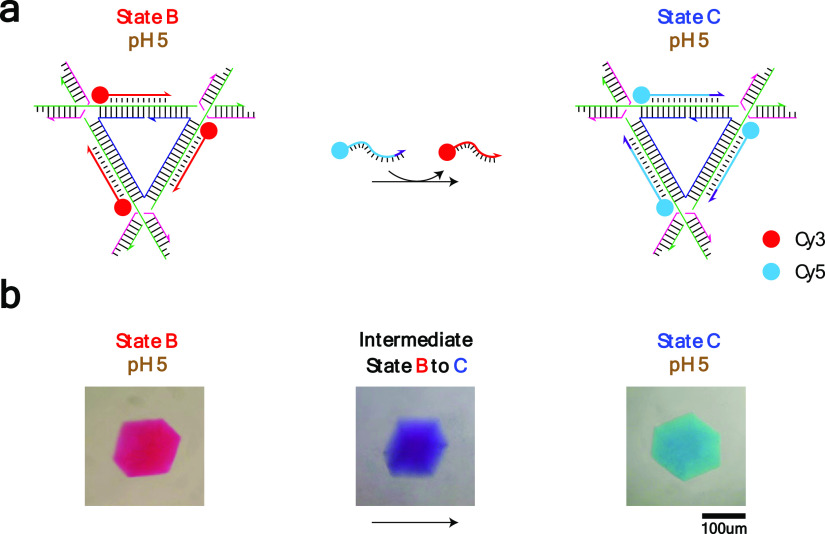
TFO length difference-induced TFO displacement. (a) Schematic drawing of the strategy: The 13-nt Cy5-bearing TFO displaces the 11-nt Cy3-bearing TFO using the toehold on the duplex edge of the triangle at pH 5.0. (b) Crystal images: Corresponding to the scheme shown above, different lengths of TFOs induced TFO displacement from state B (red) with a Cy3-bearing 11-nt TFO to state C (blue) a Cy5-bearing 13-nt TFO. The crystal image above the transition arrow shows an intermediate state (purple).

Here, we have demonstrated the formation and displacement of TFOs within the DNA tensegrity triangle in solution by using nondenaturing polyacrylamide gel electrophoresis (PAGE) (Figure 2) and in its crystal by using TFO strands bearing a variety of optical dyes that report the presence or absence of the strand from the crystalline framework (Figures 3, 4, and 5). The modified strands color the crystals in designated and dependable ways, thereby indicating the binding or removal of individual TFOs, which also could be demonstrated by X-ray crystallography.

### Triplex Formation and Displacement by pH Adjustment

Perhaps the simplest means to reconfigure triplex formation is through pH adjustment due to the pH dependence of the C^+^·GC triplet. Before undertaking crystal experiments, we examined triplex formation in solution at both pH 5 and 7 by nondenaturing PAGE (Figure 2, lanes 1–4). The tensegrity triangle was first annealed before addition of the TFOs at a 1:1 ratio of TFO to binding site, and the complexes were left to equilibrate overnight. Samples were prepared both at pH 7 (Figure 2a) and at pH 5 (Figure 2b). In both cases, and in the absence of TFO, the triangle ran as a single band with the expected mobility (lane 3). With the addition of the TFO, the mobility of the complex was shifted on account of the TFO binding to the triangle but, as expected, only when the complexes were formed at pH 5 (lane 4) and not pH 7.

We then used this triplex-based system to create reconfigurable self-assembled 3D DNA crystals that respond to pH change (Figure 3a). To observe the binding and release of the TFOs within the crystals, we used colored dyes attached covalently to specific TFOs (Figure 3b). Crystals were first grown at pH 7 using the hanging drop vapor diffusion method against a reservoir containing 600 μL of 1.75 M ammonium sulfate at room temperature, and the crystals grew to the expected size and morphology (state A). We then adjusted the pH of the crystal by removing a crystal and placing it in a drop at pH 5 before addition of a Cy3-bearing 11-nt TFO to the crystal drop and leaving for 2 days. TFO penetrance and binding to the individual triangles of the crystal were indicated by the color change of the crystal from clear (state A) to red (state B). To demonstrate the reversible nature of the interaction, we then transferred the crystal back to a drop at pH 7 and the TFO can be seen to dissociate from the triangle, as indicated by the color change of the crystal from red (state B) back to clear (state A). To further demonstrate the ability to introduce a second TFO into the system, we again decreased the crystal pH to 5 and added a Cy5-bearing 13-nt TFO to the solution. Again, this showed a color change from clear (state A) to cyan (state C) and pH-dependent formation of triplexes within the self-assembled crystals.

We have also included crystal images of the intermediate states between state A to B and state A to C (Figure 3b and Figure S1). Because the binding and dissociation of the TFOs start at the edge of the crystal and slowly move into the crystal center, a different color pattern of these crystals was observed. For example, the edge of the crystal was red and the center was clear in the intermediate states from state A (clear) to B (red). However, the color pattern was reversed in the intermediate states from state B to A.

### Triplex Formation and Displacement by Toehold-Mediated Strand Invasion

Strand displacement reactions in DNA complexes are often initiated at single-stranded regions (a “toehold”) that are designed to be complementary to an “invading” strand and progress through a branch migration process. The invading strand forms more base pairs with the toehold-containing strand, thus displacing its original complement.^22^ Furthermore, strand displacement processes in triple helical context have also been reported.^23−25^ Here, a similar strategy was used to control triplex-based reconfiguration within a crystal (Figure 4a). We first examined the triplex strand displacement reaction in solution on isolated tensegrity triangles (Figure 2a). An 11-nt TFO containing a 4-nt toehold was first bound to the triangle at pH 5 as above and followed by the addition of the full Watson–Crick complement of the TFO sequence to the solution at a 1:1 ratio. The strand displacement process can be seen to have occurred due to (a) duplex formation of the TFO with its complement and (b) the faster mobility for the DNA complex as the TFO was displaced from the triplex region of the triangle (lane 5).

To demonstrate the toehold-mediated strand displacement of TFOs within self-assembled crystals, we modified one of the component strands of the tensegrity triangle motif to contain a fluorescein dye (Figure 4b). Crystals assembled by using the fluorescein-modified strand resulted in yellow-colored crystals (state A′). To monitor triplex formation and displacement, we added the Cy3-labeled 11-nt TFO with a toehold to the crystal in state A′ and observed triplex formation as indicated by the color change of the crystal from yellow (state A′) to coral (state B′). We subsequently added the full Watson–Crick complement to the solution, and the TFO was displaced, resulting in a crystal color change back to yellow (state A′). To demonstrate the ability to introduce/remove a second TFO sequence into the crystal system, we next added a Cy5-labeled 13-nt TFO with a toehold to the crystal. As expected, this resulted in a green-colored crystal (state C′) that could also be displaced using its Watson–Crick complement strand.

### Displacement of Shorter TFOs by Longer TFOs

Our last strategy to achieve triplex-based reconfiguration exploited a TFO length difference to initiate displacement (Figure 5a). It relies on the fact that a shorter TFO bound to the triangle is displaced by a longer TFO that uses unoccupied base pairs on the duplex edge as its toehold (i.e., the Hoogsteen face of the base pairs). We first used nondenaturing PAGE to examine the TFO strand displacement reaction in solution, but it was not possible to determine strand displacement due to the resolution of the gel (Figure 2a, lane 5). Nevertheless, we undertook the same experiments with the DNA crystals and, as before, assembled the crystals at pH 5 with a short Cy3-labeled 11-nt TFO bound to the DNA triangle and successful triplex formation can be seen as a red color change of the crystal (state B) (Figure 5b). We next added a longer Cy5-labeled 13-nt TFO to the crystal drop, which led initially to an intermediate state with a purple color before the displacement of the 11-nt TFO by the 13-nt TFO and a final color of cyan (state C).

### X-ray Diffraction of Crystals in States A and C

In addition to the polyacrylamide gel mobility analysis and the color-changing assay of DNA crystals, we also performed preliminary X-ray diffraction experiments on the 3D DNA crystals containing TFOs. Crystals diffracted to ∼7 Å, consistent with earlier results we obtained for the 3-turn-per-edge triangles.^15−17^ The electron density from this preliminary data showed that the DNA triangles self-assembled just like we designed with additional electron density, indicating probable locations for TFO binding (Figure S2). We were unable to see the whole density of TFO in the refined structure due to three possibilities: (a) the resolution of the crystal is low, (b) the occupancy of TFO is low, and (c) triple helices are very dynamic, particularly at the TFO ends. Nevertheless, the underlying structure of the crystal was not perturbed in the presence of the TFO. With the recent efforts to improve self-assembled DNA crystal resolution through biological production of component strands^26^ and modification of strand termini,^27^ as well as the design of TFOs with higher affinity,^28−31^ we are approaching the success of crystallographic structure determination for biological guests contained within self-assembled lattices. While the structural aim is still being pursued, realization of dynamic strand displacement processes within these self-assembled crystals will enable the operation and programming of further nanomechanical devices.

## Discussion

The advantage of using triple helix formation for strand displacement reactions is that both duplexes and triplexes are rigid structures. During the process of displacing the TFOs, the stability and integrity of the DNA tensegrity triangle and crystal will not be substantially affected. This is in contrast to displacing one of duplex strands, which is widely used in the toehold-mediated strand displacement. To that end, we have demonstrated that it is not only possible to bind a TFO to the lattice but also to displace it from its location within the crystal. The strand displacement reactions described in the crystals were observed at their end points (typically after 1–2 days). In our earlier work with a 2-turn DNA triangle motif, we calculated strand displacement kinetics within the crystal to be dependent on the invading strand concentration as well as the crystal size.^32^ In a triplex context, we expect a similar effect of the invading TFO concentration on the kinetics of strand displacement. In the present study, we have utilized buffers containing magnesium ions to stabilize the triplexes within our crystals. However, other ions can also promote triplex formation, for example, copper or silver.^11,33−36^ Interestingly, the latter act to reduce the pH dependence of unmodified TFOs by replacing the N3 proton in the C^+^·GC triplet. If applied to our system, this would not only enable strand displacement reactions at higher pH values but also the site-specific incorporation of silver ions within the crystal lattice, which might aid the diffraction analysis of the crystals. Since triplex formation has already been exploited for the programmable positioning of non-nucleic acid components within DNA nanostructures by their attachment to the third strand, this approach will be useful for applications that require the addition, removal, or exchange of components at a later stage.

## Supporting Information Available

Materials and methods; sequences of the component strands; data collection; color changing of one crystal for the whole cycle; preliminary electron density of the state B crystal (PDF)

## Notes

In memory of Prof. Nadrian C. Seeman, who passed away during the preparation of this manuscript on November 16, 2021.
