# Supplementary material for: The Formation and Displacement of Ordered DNA Triplexes in Self-Assembled Three-Dimensional DNA Crystals
Source: J Am Chem Soc. 2023 Feb 2;145(6):3599–605. doi: 10.1021/jacs.2c12667 (PMC10032566; doi:10.1021/jacs.2c12667)
Supplement: Supplementary file 1 — ja2c12667_si_001.pdf [file ja2c12667_si_001.pdf]

Supplementary Information

The Formation and Displacement of Ordered DNA Triplexes in Self-Assembled 3D DNA Crystals

Yue Zhao[1], Arun Richard Chandrasekaran[2], David A. Rusling[3]*, Karol Woloszyn,[1] Yudong Hao[1], Carina Hernandez[1], Simon Vecchioni[1], Yoel P. Ohayon[1], Chengde Mao[4], Nadrian C. Seeman[1#] and Ruojie Sha[1]*

[1]Department of Chemistry, New York University, New York, NY 10003, USA

[2]The RNA Institute, University of Albany, State University of New York, Albany, NY 12222, USA.

[3]School of Pharmacy and Biomedical Sciences, University of Portsmouth, Portsmouth, PO1 2DT, UK

[4]Department of Chemistry, Purdue University, West Lafayette, IN 47907, USA

[#]Deceased

*Corresponding authors: ruojie.sha@nyu.edu (R.S.), David.Rusling@port.ac.uk (D.A.R.)

Table of contents

1. Materials and methods
2. Sequences of the component strands
3. Data collection
4. Color changing of one crystal for the whole cycle
5. Preliminary electron density of State B crystal

Materials and methods

Oligonucleotide synthesis and purification. DNA sequences were designed using the program SEQUIN.[1] Strands labeled with Cy3, Cy5 and Fluorescein (Glen Research phosphoramidites) were synthesized using standard phosphoramidite techniques on an Applied Biosystems 394 DNA synthesizer; other strands were purchased from Integrated DNA Technologies (Coralville, IA). Strands were purified by denaturing gel electrophoresis.

Triangle assembly

Oligonucleotides for each tile were mixed stoichiometrically in TA-Mg buffer (40mM tris-acetate containing 15mM magnesium acetate), pH 5.0 or pH 7.0, and annealed using the following protocol: 90 °C for 5 minutes, 65 °C for 20 minutes, 45 °C for 20 minutes, 37 °C for 30 minutes and 20 °C for 30 minutes. The TFOs were added after tile assembly and left to equilibrate overnight at 4 °C.

Polyacrylamide gel electrophoresis (PAGE) analysis

The complexes assembled by the DNA triangles (4 μM) and TFOs (12 μM) were run on a non-denaturing 8% polyacrylamide gels in the appropriate TA-Mg buffer at 4 °C and then visualized by staining the gel with Stains-All (Sigma Aldrich).

Crystallization

4 μL of assembled DNA triangle solution was mixed with 4 μl crystallization buffer (40 mM appropriate TA-Mg buffer and 600 mM ammonium sulfate) and then incubated against a 600 μl reservoir of 1.75 M ammonium sulfate in a hanging-drop setup at 19 °C for 2 days.

Controlling crystal transitions

To achieve the reversible pH induced transitions, a crystal from the drop at pH 7 was transferred into a drop at pH 5 and incubated at 19 °C for 24 hours. Then Cy3- or Cy5-labeled TFO (molar ratio for TFO:TFO binding sites = 1.5:1) was introduced into the pH 5 drop and further incubated at 19 °C for 2 days. The crystal was then transferred back to the pH 7 drop for TFO detachment.

To displace the short TFO with the long TFO at pH 5.0, the 13-nt Cy5-labeled TFO was introduced into the drop with the 11-nt Cy3-labeled TFO crystal (molar ratio for Cy3-TFO:Cy5-TFO = 1:1.5) and further incubated at 19 °C for 2 days. To achieve the specific sequence's recognition at pH 5.0, the Cy3- or Cy5-TFO was introduced into the drop and further incubated at 19 °C for 2 days. The strands complementary to the Cy3- or Cy5-TFO were then introduced (molar ratio TFO:complementary = 1:1.5) and further incubated at 19 °C for 2 days.

X-ray diffraction data collection and processing

Crystals were transferred to a 4× crystallization buffer containing cryogenic solvent of 30% glycerol and were frozen by immersion into liquid nitrogen. X-ray diffraction data were collected on beam lines 17ID and 19ID at the Advanced Photon Source (Argonne National Laboratory, Lemont, Illinois, USA) were processed using the programs of *HKL-2000*,[2] *PHENIX*[3] and *COOT*.[4]

Structure analysis

Using the three-turn-per-edge tensegrity triangle[5] as the search model, molecular replacement (MR) and refinement were performed on the datasets of the TFO bound crystal. The (2Fo-Fc) composite maps show the density of the TFO attached to the triangle.

**Three-turn symmetric triangle**	
T1 (14mer)	5'-TCT GAT GTG GCT GC-3'
T1_F (14mer)	5'-TCT GAX GTG GCT GC-3' (where X is FAM-C6-dT)
T2 (31mer)	5'-GAG CAG CCT GAA GAA AGA AGA GAG GAC ATC A-3'
T3 (51mer)	5'-TTC TTT CTT CAC CTC TCT TCT TTC TTC ACC TCT CTT CTT TCT TCA CCT CTC-3'
**Triplex-forming oligonucleotides (TFOs)**	
TFO1 (11mer)	5'-Cy3-TTC TTT CTT CT-3'
TFO2 (13mer)	5'-Cy5-TTC TTT CTT CTC T-3'
TFO3 (14mer)	5'-Cy3-TTC TTT CTT CTT CG-3'
TFO4 (16mer)	5'-Cy5-TTC TTT CTT CTC TGA C-3'
TFO3_Comp (14mer)	5'-CGA AGA AGA AAG AA-3'
TFO4_Comp (16mer)	5'-GTC AGA GAA GAA AGA A-3'

Table S1. Sequences of the Component Strands. The three-turn symmetric tile is composed of three strands: T1, T2 and T3 (state A). T1_F is the similar sequence as T1 strand with fluorescein modified for observation purpose of microscopic crystal (state A'). The TFOs sequences are indicated as TFO1 and TFO 2 for state B and state C respectively, TFO 3 and TFO 4 for state B' and state C' respectively. TFO3_Comp and TFO4_Comp are the complementary strands for the toehold displacement for TFO3 in state B' and TFO4 in state C' respectively.

**X-ray data collection**	
Wavelength (Å)	1.100
Space Group	R3
Cell dimensions	
a = *b* = *c* (Å)	101.4
$\alpha = \beta = \gamma$ (°)	111.9
Resolution (Å)	83.98 – 5.30<br>(6.01 – 5.30)
Ellipsoidal diffraction limits[a] (Å)	5.21, 5.21, 7.11
CC(1/2)	0.997 (0.189)
Completeness ellipsoidal (%)[a]	81.3 (42.0)
Redundancy	9.7 (10.3)

*Values in parentheses represent the highest resolution shell.

[a]Data scaling performed with ellipsoidal cutoff using the STARANISO server (Global Phasing), with severe anisotropy observed in all crystals along the three-fold axis.[6]

Table S2. X-ray data analysis of crystal in State B.

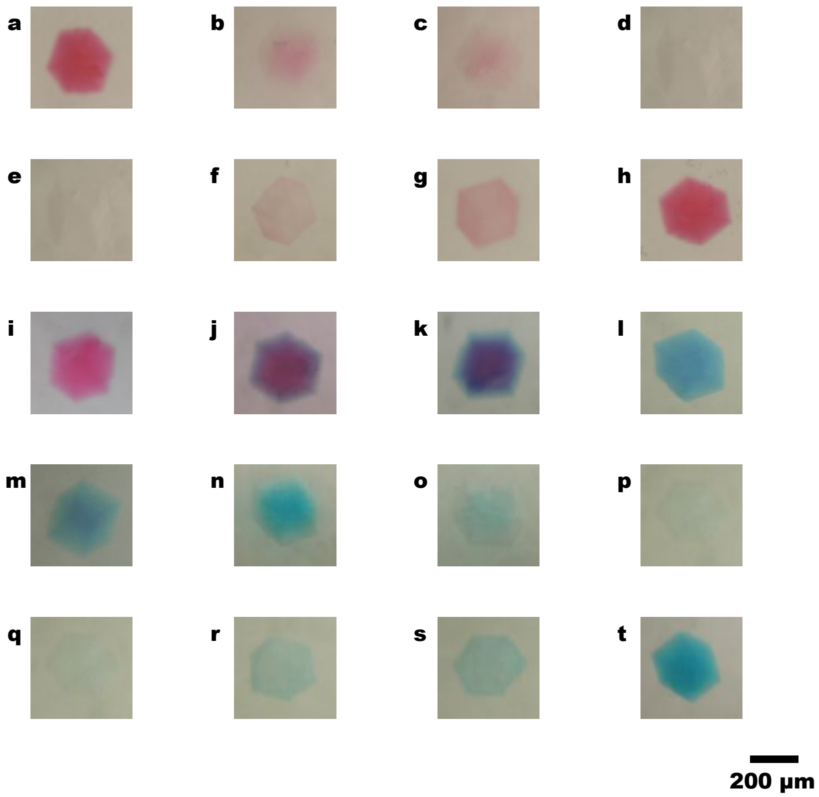

Figure S1. Color changing of one crystal for the whole cycle corresponding to Figure 3 and Figure 5. a-d, State B to state A corresponding to Figure 3; e-h, State A to state B corresponding to Figure 3; i-l, State B to state C corresponding to Figure 5; m-p, State C to state A corresponding to Figure 3; q-t, State A to state C corresponding to Figure 3. Scale bar is 200 microns.

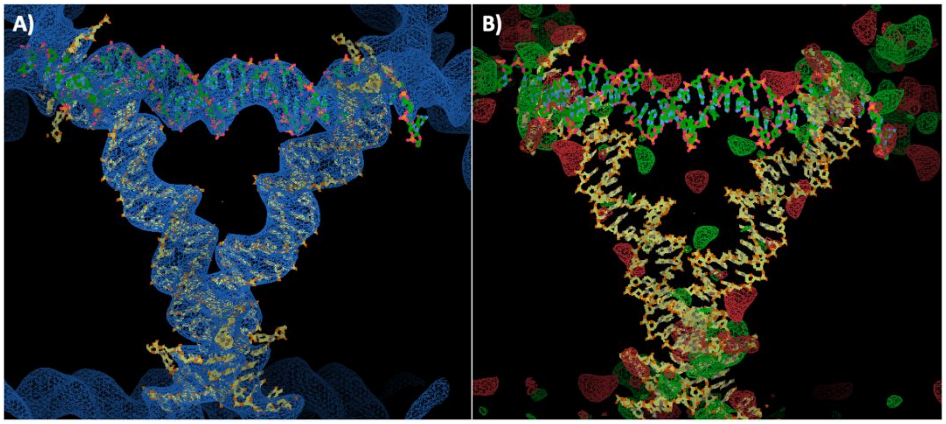

Figure S2: Electron density of State B crystals refined using reported 3-turn structure 3UBI. **A)** $2F_o$-F_c contoured at 2.2 rmsd and **B)** F_o-F_c contoured at 3.5 rmsd do not possess significant density to allow refinement of a major-groove-bound TFO.

References

(1) Seeman, N. C. De Novo Design of Sequences for Nucleic Acid Structural Engineering. *Journal of Biomolecular Structure and Dynamics* **1990**, *8* (3), 573–581. https://doi.org/10.1080/07391102.1990.10507829.

(2) Otwinowski, Z.; Minor, W. [20] Processing of X-Ray Diffraction Data Collected in Oscillation Mode. In *Methods in Enzymology*; Macromolecular Crystallography Part A; Academic Press, 1997; Vol. 276, pp 307–326. https://doi.org/10.1016/S0076-6879(97)76066-X.

(3) Adams, P. D.; Grosse-Kunstleve, R. W.; Hung, L.-W.; Ioerger, T. R.; McCoy, A. J.; Moriarty, N. W.; Read, R. J.; Sacchettini, J. C.; Sauter, N. K.; Terwilliger, T. C. PHENIX: Building New Software for Automated Crystallographic Structure Determination. *Acta Cryst D* **2002**, *58* (11), 1948–1954. https://doi.org/10.1107/S0907444902016657.

(4) Emsley, P.; Cowtan, K. Coot: Model-Building Tools for Molecular Graphics. *Acta Cryst D* **2004**, *60* (12), 2126–2132. https://doi.org/10.1107/S0907444904019158.

(5) Nguyen, N.; Birktoft, J. J.; Sha, R.; Wang, T.; Zheng, J.; Constantinou, P. E.; Ginell, S. L.; Chen, Y.; Mao, C.; Seeman, N. C. The Absence of Tertiary Interactions in a Self-Assembled DNA Crystal Structure. *Journal of Molecular Recognition* **2012**, *25* (9), 494–494. https://doi.org/10.1002/jmr.2214.

(6) Tickle, I.J., Flensburg, C., Keller, P., Paciorek, W., Sharff, A., Vonrhein, C., Bricogne, G. (2018). STARANISO (http://staraniso.globalphasing.org/cgi-bin/staraniso.cgi). Cambridge, United Kingdom: Global Phasing Ltd.
